# Supplementary material for: Time-programmable drug dosing allows the manipulation, suppression and reversal of antibiotic drug resistance in vitro
Source: Nat Commun. 2017 Jun 8;8:15589. doi: 10.1038/ncomms15589 (PMC5472167; doi:10.1038/ncomms15589)
Supplement: Supplementary Information — Supplementary figures, supplementary tables, supplementary methods and supplementary references. [file ncomms15589-s1.pdf]

# SUPPLEMENTARY INFORMATION

**Supplementary Table 1** List of mutations observed in CHL/POL only, or CHL and POL cycling evolution experiments.

| position | genes | change | annotation | CHL | POL | POL-CHL | CHL-POL | | | |
|---|---|---|---|---|---|---|---|---|---|---|
| | | | | | | | Day 6 | Day 10 | Day 18 | Day 24 |
| 66,528 | araD | T→C | Q8R (CAG→CGG) | 68.0 | | | | | | |
| 70,289 | araB/araC | G→T | intergenic ( 241/ 98) | 70.9 | | | | | | |
| 212,943 | tilS | T→A | F205I (TTT→ATT) | 37.0 | | | | | | |
| 212,943 | tilS | T→A | F205I (TTT→ATT) | | | | 80.4 | | | |
| 366,519 | lacI | G→A | A331V (GCG→GTG) | 54.3 | | | | | | |
| 428,465 | secD → | G→A | L273L (CTG→CTA) | | 100.0 | 100.0 | | 100.0 | 100.0 | 100.0 |
| 503,429 | ybaL/fsr | C→T | intergenic ( 191/+47) | 68.9 | | | | | | |
| 585,876 | ompT ← / → pauD | IS1 (–) +9 l | intergenic ( 243/ 173) | | | 100.0 | | | | |
| 705,013 | nagE | T→C | L357S (TTG→TCG) | 62.5 | | | | | | |
| 707,537 | glnS | C→T | P482L (CCA→CTA) | | 41.8 | | | 41.8 | 50.5 | 37.5 |
| 883,553 | ybjG ← / → mdfA | IS30 (+) +2 | intergenic (_165/_119) | | 100.0 | | 100.0 | 100.0 | 100.0 | 100.0 |
| 883,596 | ybjG/mdfA | T→G | intergenic (_208/_77) | 19.1 | | | | | | |
| 883,646 | ybjG/mdfA | C→T | intergenic (_258/_27) | | | | | | 46.2 | 53.8 |
| 883,647 | ybjG/mdfA | G→A | intergenic ( 259/ 26) | | | | | | 38.2 | |
| 883,661 | ybjG/mdfA | C→A | intergenic ( 273/ 12) | 29.5 | | | | | | |
| 1,337,394 | acnA | A→G | S522G (AGC→GGC) | 65.6 | | | | | | |
| 1,619,300 | marR | A→. | coding (181/435 nt) | 29.9 | | | | | | |
| 1,652,331 | intQ | T→C | pseudogene (781/1158 nt) | 66.9 | | | | | | |
| 2,406,196 | lrhA | .→C | coding (446/939 nt) | 22.6 | | | | | | |
| 2,492,470 | frc ← | G→T | T262N (ACC→AAC) | | 100.0 | 100.0 | | 100.0 | 100.0 | 100.0 |
| 2,844,010 | hycG | G→T | R67R (CGC→CGA) | 77.7 | | | | | | |
| 3,110,421 | pheV | C→A | noncoding (56/76 nt) | 64.0 | | | | | | |
| 3,703,260 | dppD | G→A | L197L (CTG→TTG) | 29.4 | | | | | | |
| 4,035,727 | rrsA | A→T | noncoding (197/1542 nt) | 57.4 | | | | | | |
| 4,093,770 | rhaD | C→T | G168D (GGC→GAC) | 64.9 | | | | | | |
| 4,161,248 | fabR | G→T | G42V (GGC→GTC) | 65.8 | | | | | | |
| 4,164,123 | btuB | C→G | A162G (GCA→GGA) | 67.2 | | | | | | |
| 4,296,190 | gltP/yjcO | A→G | intergenic (+396/+246) | 17.1 | | | | | | |
| 4,332,995 | basS | C→G | R93P (CGC→CCC) | | 53.5 | | | 53.5 | 50.7 | 65.7 |
| 4,333,010 | basS | A→T | V88E (GTA→GAA) | | | 25.7 | | | | |
| 4,333,050 | basS ← | (TAGCGTC | coding (223/1092 nt) | | 100.0 | | | 100.0 | 100.0 | 100.0 |
| 4,333,232 | basS | A→C | L14R (CTG→CGG) | | | 64.4 | | | | |
| 4,474,834 | bdcR/tabA | A→G | intergenic (+117/ 28) | 66.0 | | | | | | |
| 4,585,480 | hsdR | G→A | Q428* (CAA→TAA) | 72.3 | | | | | | |
| 4,602,509 | yjjP | C→T | W117* (TGG→TAG) | 71.0 | | | | | | |
| 4,616,669 | yjjI | G→T | L133I (CTA→ATA) | 64.1 | | | | | | |

**Supplementary Table 2** The collateral sensitivity and cross resistance profile of resistant strains used for theoretical simulations. See also Supplemetary Fig. 6.

**Antibiotic tested**

| Strain resistance | POL | CHL | NIT | NAL | KAN | AMP | RIF | TET |
|---|---|---|---|---|---|---|---|---|
| **POL** | 5.351852 | -0.54167 | 0.354167 | -1.03819 | 0.142361 | -0.39583 | -1 | 0.5 |
| **CHL** | 0.768519 | 4.666667 | 0.4375 | 2.461806 | 0.225694 | 1.854167 | 1 | 4.25 |
| **NIT** | 1.231481 | 0 | 4.916667 | 0.173611 | 0.083333 | 0.152778 | 3.5 | 0 |
| **NAL** | 0.814815 | 0.916667 | 0.708333 | 7.506944 | 0.166667 | 0.736111 | 0.75 | 1 |
| **KAN** | -0.18519 | -0.33333 | 0 | -0.24306 | 2.833333 | 0.152778 | 3.25 | 0 |
| **AMP** | 0.481481 | 0.166667 | 0.041667 | -0.24306 | 0.75 | 5.027778 | 1 | 0 |
| **TMP** | -1.11111 | 1 | 1 | 0.833333 | 0.666667 | 1 | 0.5 | 0.5 |
| **RIF** | 1.138889 | 0.75 | 0 | 0.416667 | -0.20833 | -0.25 | 8 | 0.125 |
| **TET** | 0.388889 | 4 | 1 | 2.666667 | 0.291667 | 2.5 | 1 | 4.875 |

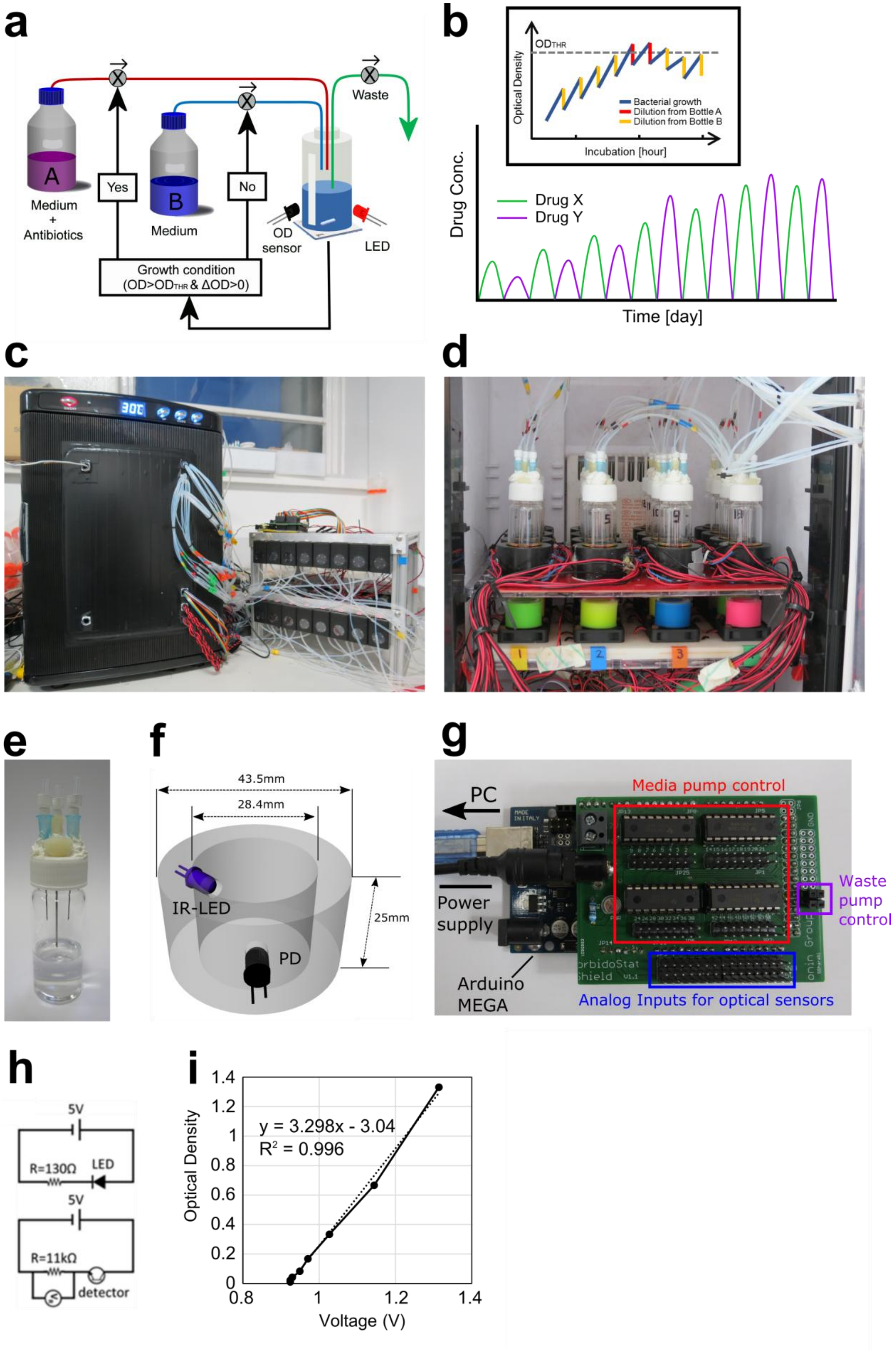
a
A
Medium
+
Antibiotics
B
Medium
Yes
No
Waste
OD
sensor
LED
Growth condition
(OD>ODTHR & ΔOD>0)
b
ODTHR
Optical Density
Bacterial growth
Dilution from Bottle A
Dilution from Bottle B
Incubation [hour]
Drug Conc.
Drug X
Drug Y
Time [day]
c
d
e
f
43.5mm
28.4mm
IR-LED
25mm
PD
g
PC
Media pump control
Power
supply
Waste
pump
control
Arduino
MEGA
Analog Inputs for optical sensors
h
5V
R=130Ω
LED
5V
R=11kΩ
detector
i
y = 3.298x - 3.04
R² = 0.996
Optical Density
Voltage (V)
1.4
1.2
1
0.8
0.6
0.4
0.2
0
0.8
1
1.2
1.4

**Supplementary Fig. 1 Morbidostat.** The morbidostat system is an automated continuous-culture device that controls drug concentration to maintain constant growth inhibition. **a**, Schematic diagram of the morbidostat system. The device runs at a certain interval and adds either fresh medium with a high drug concentration (bottle A) or without antibiotics (bottle B), on the basis of bacterial growth detected by the optical system. The cell cultures are kept at constant volume with suction. **b**, Schematic illustration of overall antibiotic cycling and bacterial growth during a single morbidostat run (inset). Antibiotics were switched every 1/3/6 day. When the OD exceeds a threshold ($OD_{THR}$) and if the growth rate is higher than the dilution rate, fresh medium is added from bottle A. In all the other cases, fresh medium from bottle B is infused. **c**, An external view of the morbidostat system. It consists of an incubator, 32 infusion pumps, one waste pump, two Arduino for pump control and optical monitoring, and a PC. **d**, Internal view of the morbidostat system. It accommodates 16 vials for parallel experiments. All the vials are mixed by PC fan-based magnetic stirrer. The entire apparatus sits in an incubator for temperature control. **e**, Morbidostat vial. The lid accommodates two liquid infusion needles, one waste removal needle, and one ventilation hole. **f**, 3D-printed holder assembly for OD measurement. An infra-red light emitting diode (IR-LED) is used as light source and photodiode (PD) is used to detect scattered light of the cell culture medium. **g**, Top view of a custom-made shield mounted on Arduino MEGA. **h**, Circuit diagrams for the optical density monitoring.

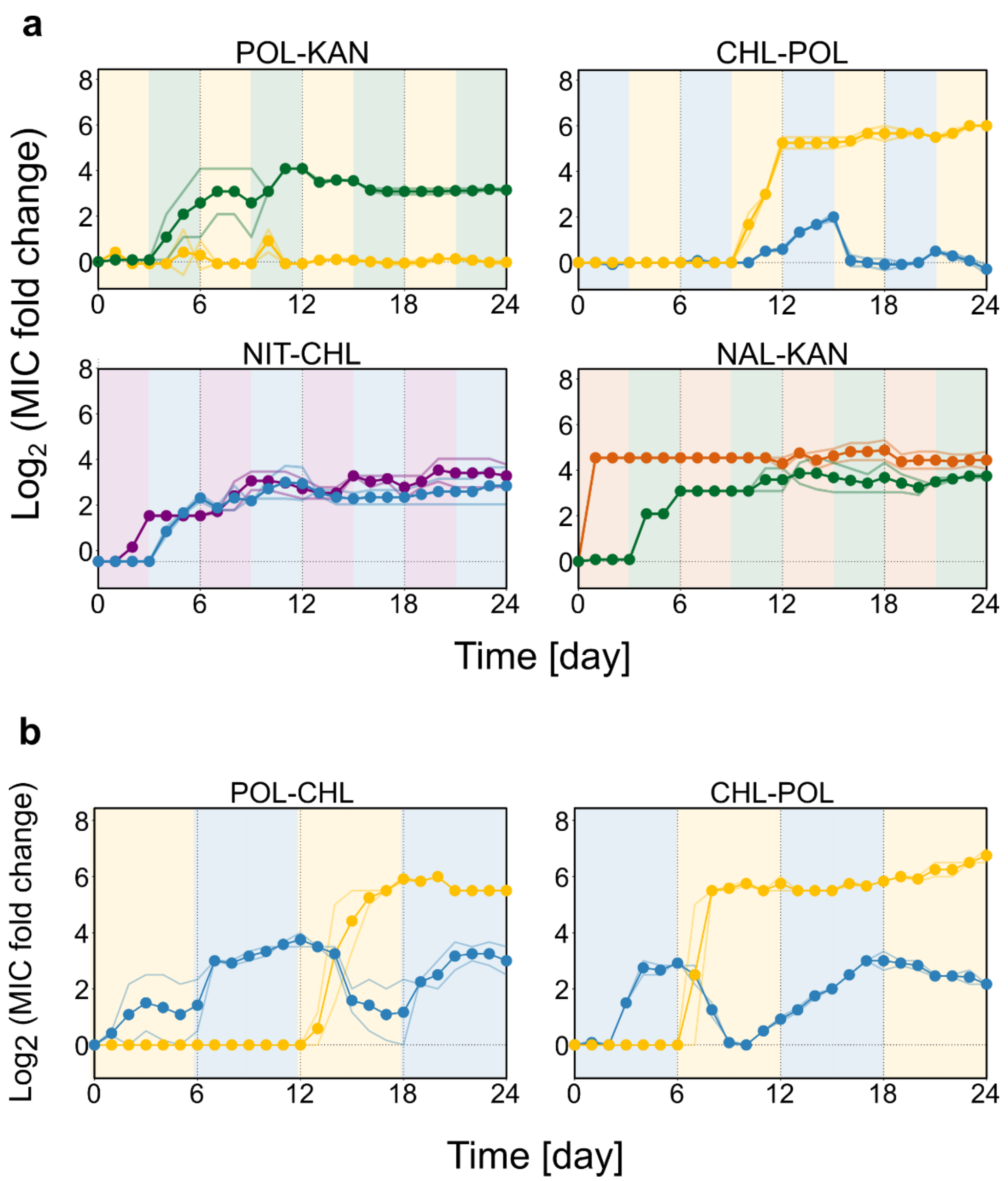


**Supplementary Fig. 2 Evolutionary trajectories under cycled antibiotic stress. a**, The antibiotics were cycled with three-day interval. Pale coloured stripe background indicates the antibiotics used. The experimental condition was same as Fig. 1 but the order of antibiotic cycling was reversed. The name of cycled antibiotics was shown on top of each plot. The name also indicates the order of antibiotics cycled. For example, POL-KAN indicates that POL and KAN were used on the first and second day, respectively, and then cycled after that. Pale coloured stripe background indicates the antibiotics used. **b**, The evolutionary trajectories of POL and CHL cycling experiments. Antibiotics were cycled with six-day interval. CHL resistance was developed during exposure to the antibiotic. However, it was reversed to zero (the MIC of wild-type strain) while the bacterial populations were exposed to POL. On the other hand, POL resistance did not show such reversible patterns.

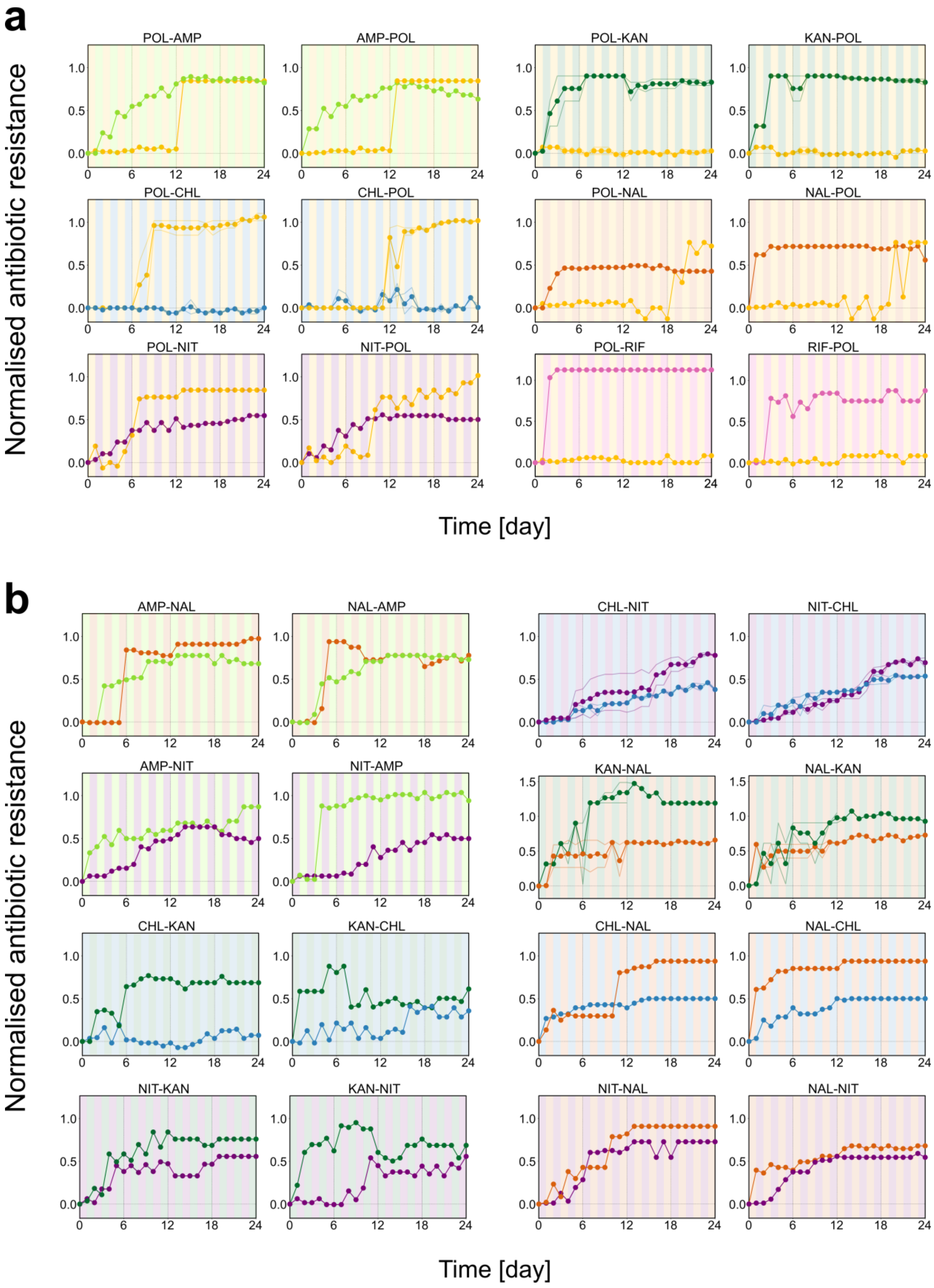
a
Normalised antibiotic resistance
POL-AMP
AMP-POL
POL-KAN
KAN-POL
POL-CHL
CHL-POL
POL-NAL
NAL-POL
POL-NIT
NIT-POL
POL-RIF
RIF-POL
Time [day]
b
Normalised antibiotic resistance
AMP-NAL
NAL-AMP
CHL-NIT
NIT-CHL
AMP-NIT
NIT-AMP
KAN-NAL
NAL-KAN
CHL-KAN
KAN-CHL
CHL-NAL
NAL-CHL
NIT-KAN
KAN-NIT
NIT-NAL
NAL-NIT
Time [day]

**Supplementary Fig. 3 Evolutionary trajectories under cycled antibiotic stress.** The antibiotics were cycled with one-day interval. The name of cycled antibiotics was shown on top of each plot. Pale coloured stripe background indicates the antibiotics used. **a**, Antibiotic cycling using POL. When cycled with AMP, CHL, and NIT, the POL resistance appeared at a relatively early stage (by the 13th day). Although the theoretical model predicted the bacterial population would result in multi-drug resistant states (Supplementary Fig. 6a), CHL-POL cycling resulted in a single drug resistance (POL resistance only). We speculate that the CHL resistance did not develop because it was easily reversed when cycled with POL (see Supplementary Fig. 2a and b), which was due to the complex and multiple adaptation pathways[1]. On the other hand, the POL resistance did not appear (with KAN and RIF) or appeared at later stage of the evolution (with NAL). In the latter case, the theoretical model predicted a single drug resistant state (NAL resistance only). This suggests that there was some additional adaptation process that alleviated the fitness costs for NAL resistance occurred during the cycling experiment (e.g. compensatory mutations[2]). Indeed, the model reproduces the experimental results when such effect was taken into account (Supplementary Fig. 6b). **b**, The antibiotics other than POL were cycled with one-day interval. In all the cases tested here, the bacterial population developed multi-drug resistance. See also Supplementary Fig. 6c for comparison with simulated evolutionary trajectories by the theoretical model.

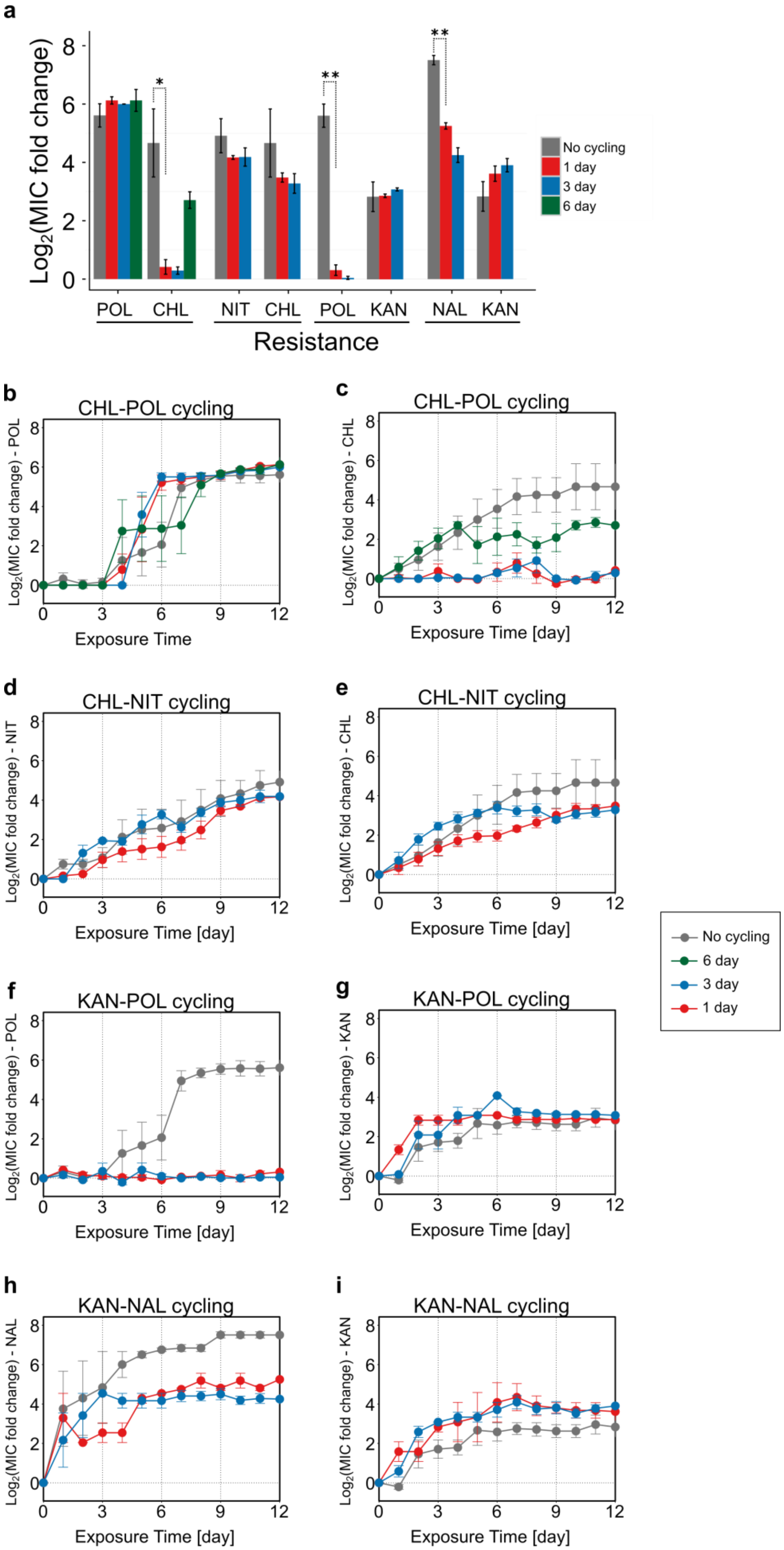
a
Log2(MIC fold change)
8
6
4
2
0
*
**
**
No cycling
1 day
3 day
6 day
POL
CHL
NIT
CHL
POL
KAN
NAL
KAN
Resistance
b
CHL-POL cycling
Log2(MIC fold change) - POL
Exposure Time
c
CHL-POL cycling
Log2(MIC fold change) - CHL
Exposure Time [day]
d
CHL-NIT cycling
Log2(MIC fold change) - NIT
Exposure Time [day]
e
CHL-NIT cycling
Log2(MIC fold change) - CHL
Exposure Time [day]
No cycling
6 day
3 day
1 day
f
KAN-POL cycling
Log2(MIC fold change) - POL
Exposure Time [day]
g
KAN-POL cycling
Log2(MIC fold change) - KAN
Exposure Time [day]
h
KAN-NAL cycling
Log2(MIC fold change) - NAL
Exposure Time [day]
i
KAN-NAL cycling
Log2(MIC fold change) - KAN
Exposure Time [day]

**Supplementary Fig. 4 Effect of cycling interval. a**, The relative antibiotic resistance was compared using the last day samples evolved under antibiotic cycling or single antibiotic condition (noted as "no cycling"). Error bars represent s.e.m. Black bars under the x axis indicate anbitibiocs pairs that were cycled (see **b-i**). Note that experiments with the different orders of cycling (e.g. CHL-POL and POL-CHL) were not distinguished here. A single asterisk indicates p ~ 0.061 (marginal) and a double asterisk p < 0.001. **b**, Evolutionary trajectories of the mean relative POL resistance of CHL and POL cycling was plotted as a function of time exposed to each antibiotic. Error bars represent s.e.m. Note that experiments with the different orders of cycling (e.g. CHL-POL and POL-CHL) were not distinguished here. **c**, CHL resistance of CHL and POL cycling. **d**, NIT resistance and **e**, CHL resistance of NIT and CHL cycling. **f**, POL resistance and **g**, KAN resistance of KAN and POL cycling. **h**, NAL resistance and **i**, KAN resistance of KAN and NAL resistance. See Fig. 1, Supplementary Fig. 2, Supplementary Fig. 3, and Supplementary Fig. 5 for the original data. Sample sizes for **a-i** are $n = 3$ for single antibiotic condition and $= 4$ for antibiotic cycling.

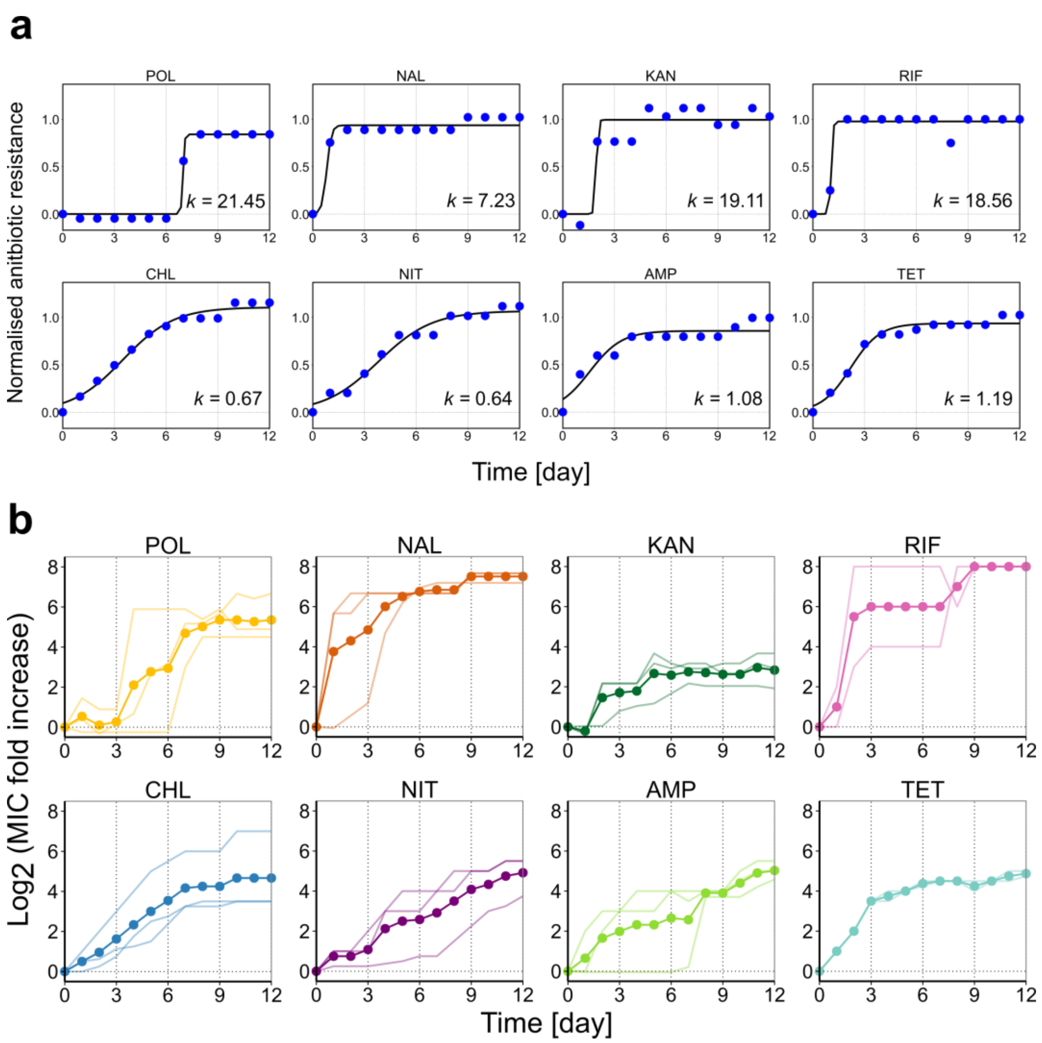


**Supplementary Fig. 5 Evolutionary trajectories under single antibiotic stress. a.** Each plot shows changes of MIC values normalised to one by the maximum MIC value of the averaged data. The name of antibiotic is shown on each plot. Representative data for each antibiotic was shown. Evolutionary trajectories were shown in blue circles and black curves indicate fitted logistic curves (See Methods). The steepness of the curve $k$ was also shown in each plot. **b.** The original unnormalised evolutionary trajectories data. Note that the same data was used in Fig. 1. The steepness of the curve $k$ and the duration of silent phase $\theta$ were used to categorise the evolutionary patterns with threshold values $\mathrm{k} = 2.5$ and $\theta = 2.5$. k and θ values (mean $\pm$ s.e.m.) for each trajectory were $\mathrm{k} = 13.7 \pm 6.45$ and $\theta = 4.64 \pm 1.34$ (POL), $5.06 \pm 1.65$ and $1.56 \pm 0.35$ (NAL), $7.11 \pm 6$ and $1.77 \pm 0.5$ (KAN), $9.48 \pm 9.1$ and $-1.45 \pm 2.31$ (RIF), $0.79 \pm 0.13$ and $0.12 \pm 0.82$ (CHL), $0.55 \pm 0.05$ and $-0.25 \pm 1.41$ (NIT), $2.28 \pm 1.5$ and $1.29 \pm 2.73$ (AMP), $1.17 \pm 0.02$ and $-0.06 \pm 0.03$ (TET). See Methods for calculation of $\theta$. Sample sizes are $n = 3$ for POL, NAL, KAN, CHL, NIT, and AMP, and $n = 2$ for RIF and TET (both biological duplicates).

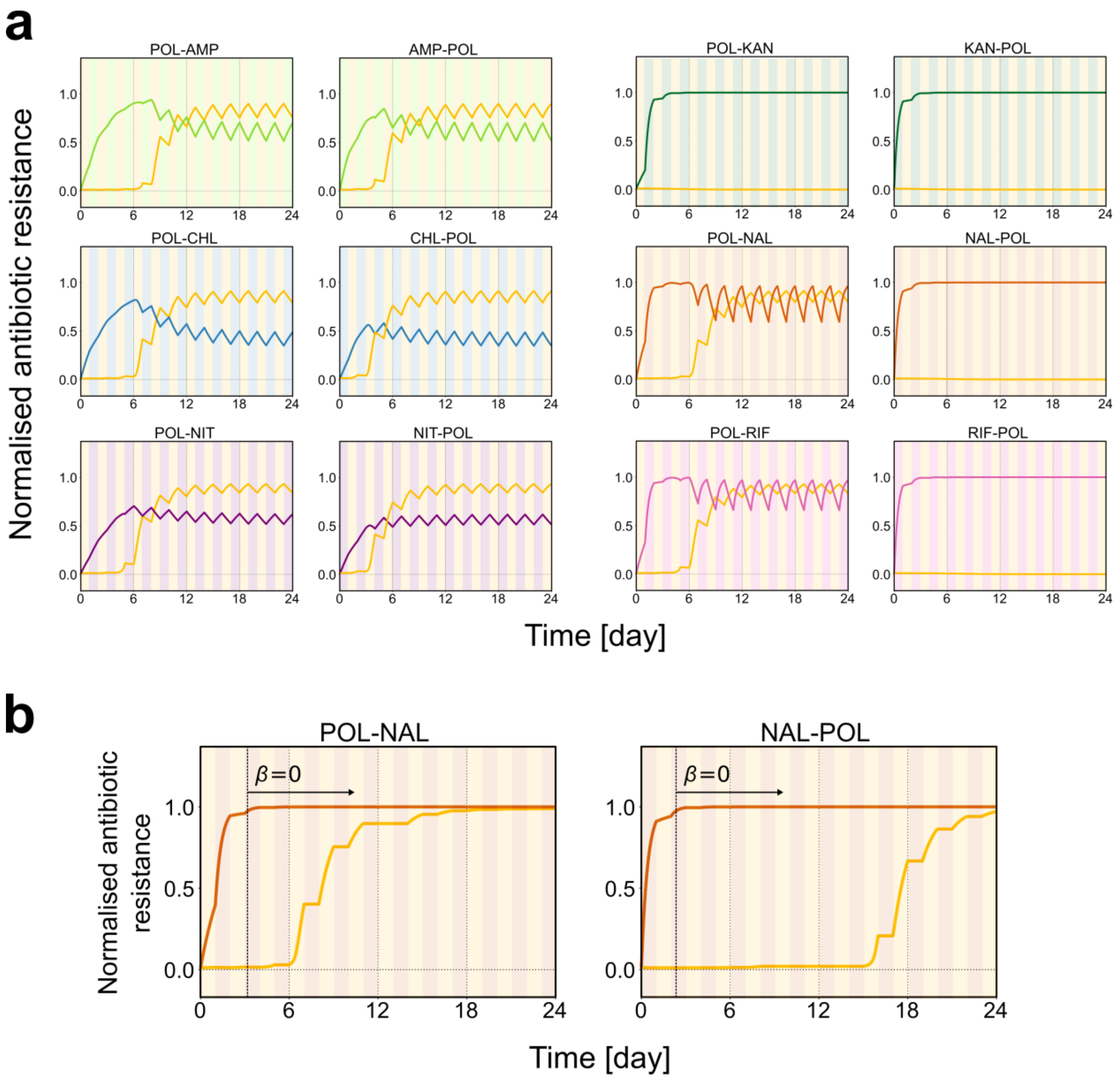
a
POL-AMP
AMP-POL
POL-KAN
KAN-POL
POL-CHL
CHL-POL
POL-NAL
NAL-POL
POL-NIT
NIT-POL
POL-RIF
RIF-POL
Normalised antibiotic resistance
Time [day]
b
POL-NAL
NAL-POL
$\beta=0$
Normalised antibiotic resistance
Time [day]

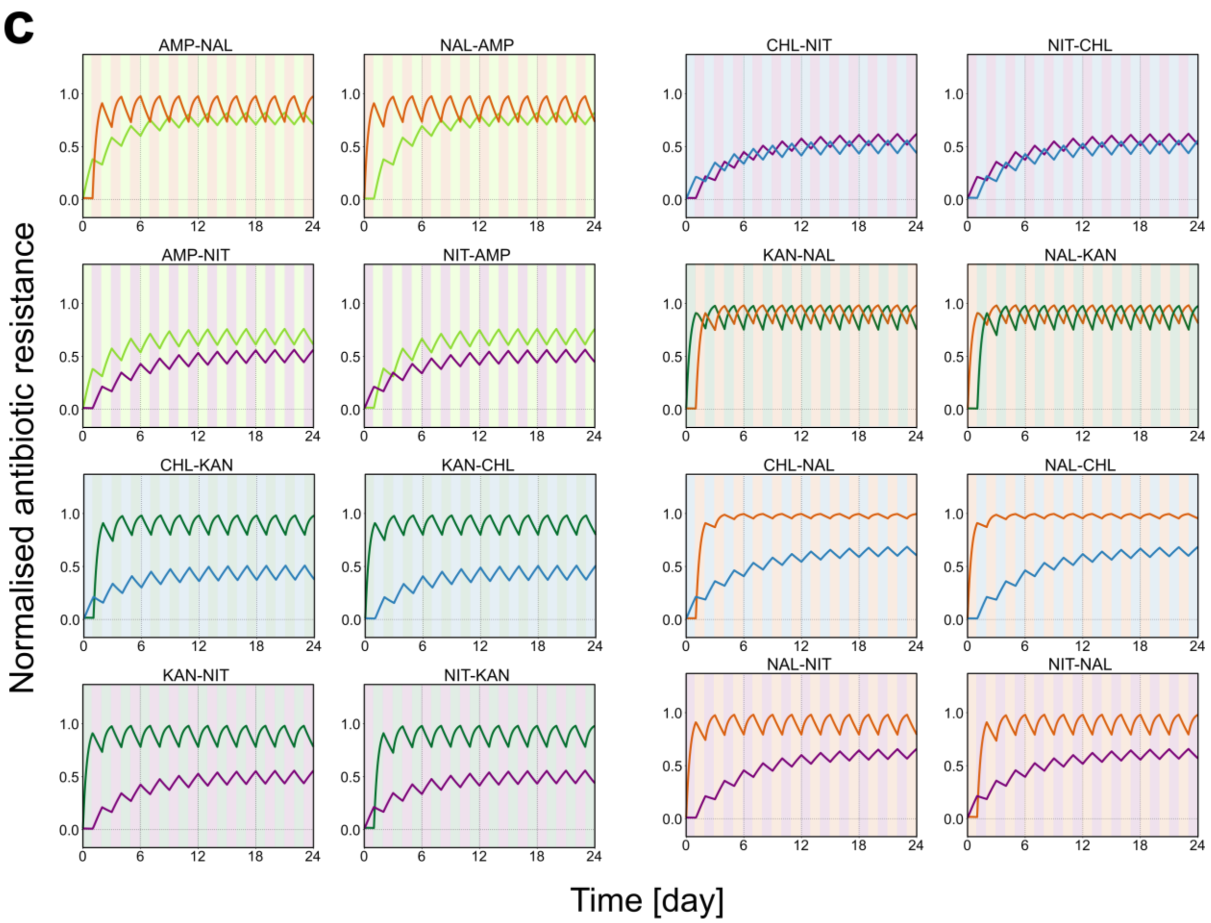


**Supplementary Fig. 6 Simulated evolutionary trajectories under cycled antibiotic stress.** The development of bacterial resistance was simulated using the theoretical model consisting of positive autoregulation and negative feedback loops. The name of cycled antibiotics was shown on top of each plot. Pale coloured stripe background indicates the antibiotics used. **a**, Antibiotic cycling using POL. With AMP, CHL, and NIT, the bacterial population developed resistance to both antibiotics (multi-drug resistant state). The POL resistance appeared around from the 6th day. On the other hand, the POL resistance was not developed with KAN, NAL, and RIF (single drug resistant state). **b**, Simulated evolutionary trajectories of NAL and POL cycling when the effect of fitness cost alleviation was taken into account. Such effect was simulated by reducing $\beta_{\mathrm{NAL-POL}}$ to zero from the middle of the second and first day (for POL-NAL and NAL-POL, respectively. Indicated by dotted vertical lines). See Supplementary Fig. 3a for comparison. **c**, Antibiotic cycling using the antibiotics other than POL with one-day interval. In all the cases, the system resulted in multi-drug resistant states.

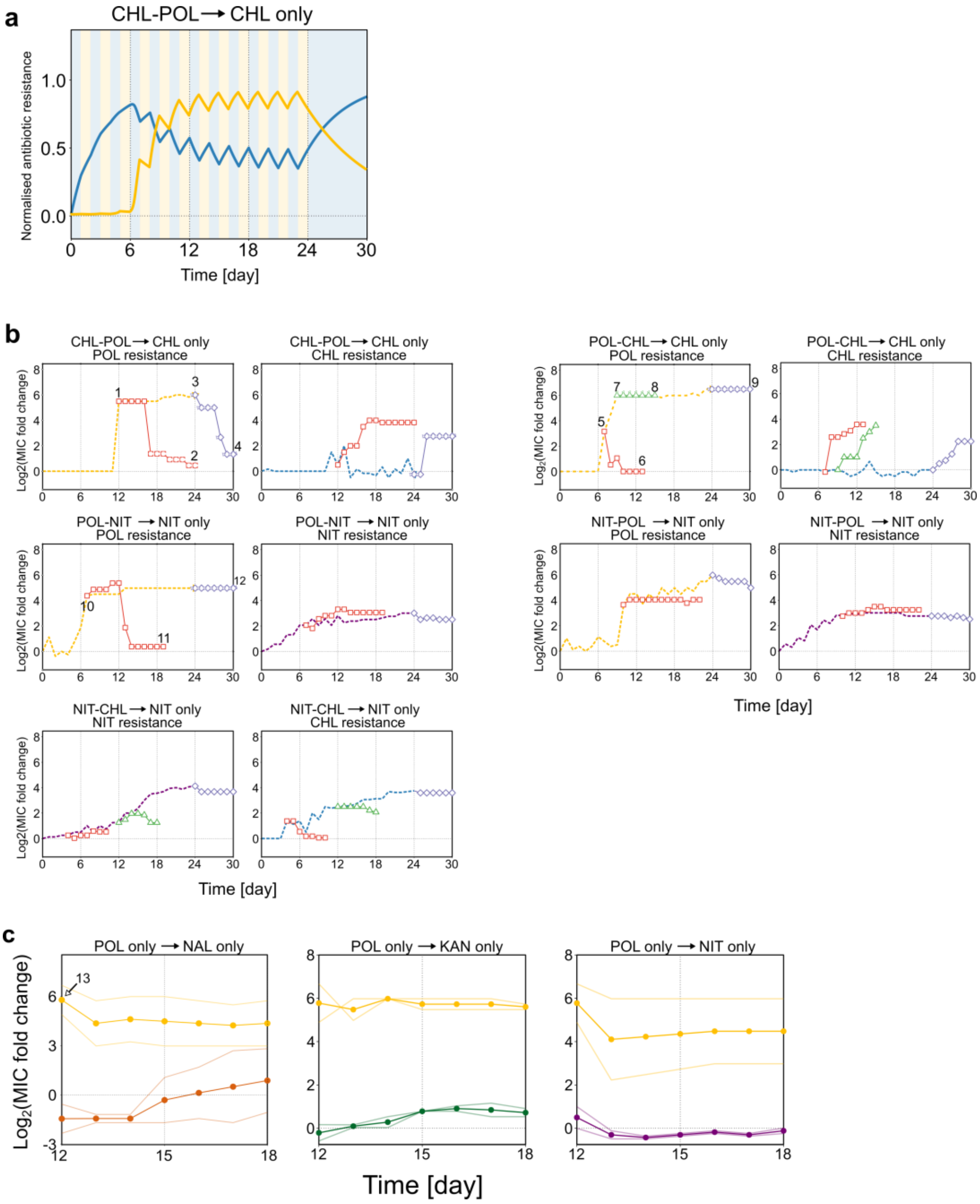


**Supplementary Fig. 7 Reversibility of antibiotic resistance. a,** Simulated results of bacterial evolution with CHL-POL cycling stress then CHL only stress. See Fig. 4a for comparison with experimental data. **b,**Evolved resistance of the bacterial population in the early stages of antibiotic cycling (indicated by red squares) was reversed by six or twelve days of consecutive stress to counteracting antibiotics (CHL or NIT). In contrast, that in the later stages was mostly not reversible (green triangles and purple diamonds). Numbers in the plots correspond to gene sequencing analysis in Supplementary Fig. 8. **c,** Evolutionary trajectories of bacterial resistance under single antibiotic stress after 12-day exposure to POL stress (see Fig. 1). No apparent reduction in the resistance level was observed either.

| Gene | 1 | 2 | 3 | 4 | 5 | 6 | 7 | 8 | 9 | 10 | 11 | 12 | 13 | Details |
|---|---|---|---|---|---|---|---|---|---|---|---|---|---|---|
| *basS* | | | 42 | | | | | | | | | 100 | 54 | R93P (CGC→CCC) |
| | | | | | | | | | | | | | 100 | Gene amplification (223/1092 nt) |
| | | | | | | | | | | | | | | V88E (GTA→GAA) |
| | | | 72 | | | | | | | | | | | L14R (CTG→CGG) |
| *frc* | | 19 | | | 73 | 100 | 75 | 69 | 100 | | | | 100 | T262N (ACC→AAC) |
| *glnS* | | | | | | | | | | | | | 42 | P482L (CCA→CTA) |
| *secD* | 42 | 15 | 16 | 79 | 89 | 89 | 100 | 100 | 100 | | 21 | | 100 | L273L (CTG→CTA) |
| *ybjG/mdfA* | | | | | | | | | | | | | 100 | intergenic (-165/-119), IS30 (+) +2 bp |
| | | | | | | | | | | | | | | intergenic (-258/-27), C→T |
| | | | | | | | | | | | | | | intergenic (-259/-26), G→A |
| Reversed? | Yes | | Yes | | Yes | | No | | No | Yes | | No | No | |

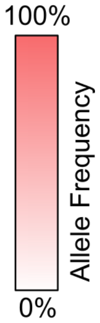


**Supplementary Fig. 8 List of mutations and frequencies in POL resistant mutants**. A number on each column corresponds to a number in Supplementary Fig. 7. Mutations were first identified by whole genome sequencing of POL resistant mutant evolved under single POL stress (See Supplementary Table 1). Mutations in the samples of reversing resistance experiments (Supplementary Fig. 7b, c) were analyzed by Sanger sequencing. Allele frequencies indicate the percentage of mutants with a specific mutation in a population, which were calculated from fluorescent signal intensities at a position of Sanger sequencing data as they reflect the frequency of a mutant in the population.

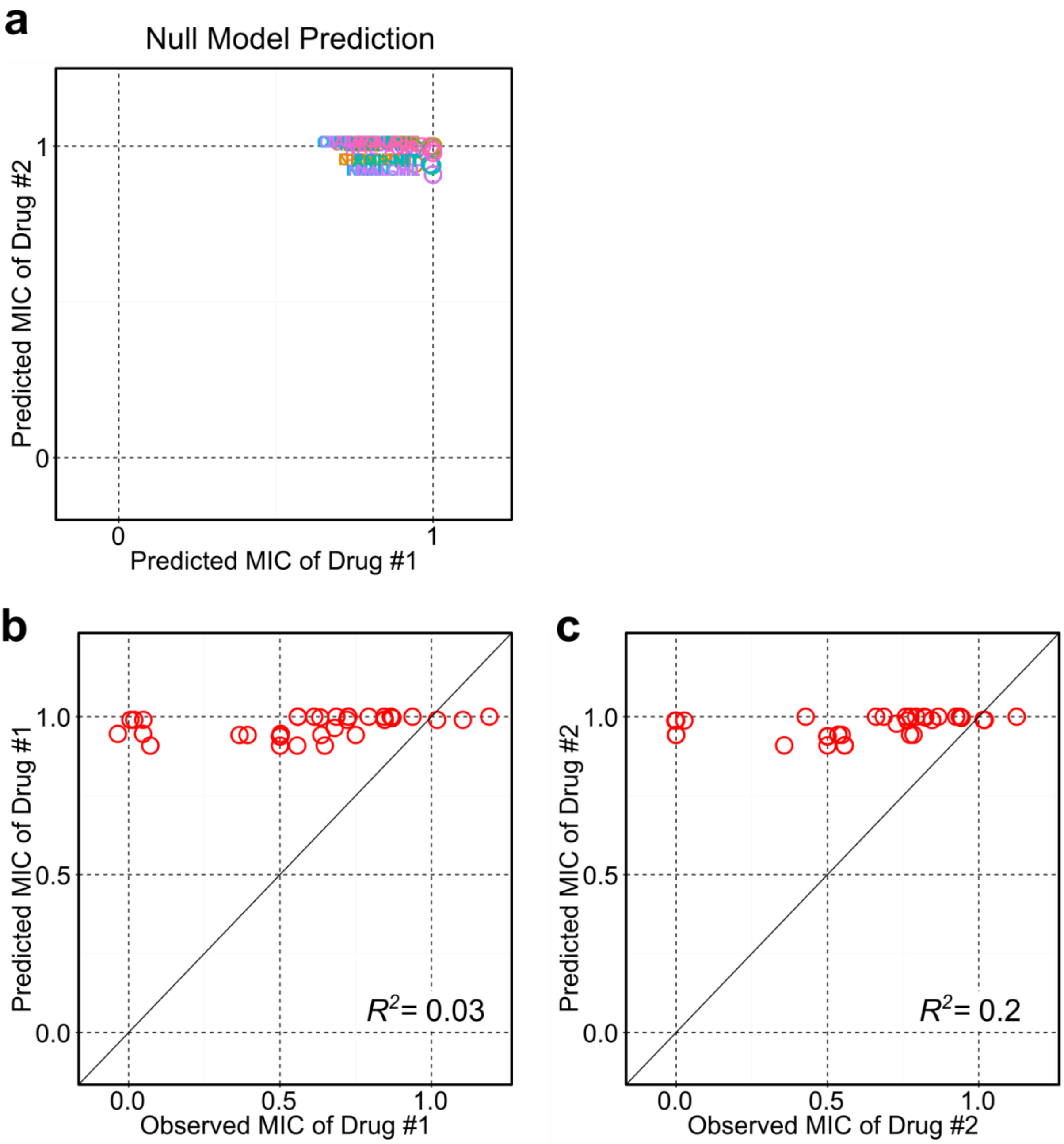


**Supplementary Fig. 9 Comparison of experimental results and null model predictions.** **a**, Scatter plot of MICs predicted by a null model where all the collateral sensitivity/cross resistance coupling parameters β were set to zero The final time point of simulated results were used in the plot. Note that the effect of fitness cost alleviation for NAL and POL cycling was incorporated in the results. **b-c**, Scatter plots of predicted MICs against observed MICs for the first drug and the second drug, respectively. Data for observed MICs are from Fig. 4a.

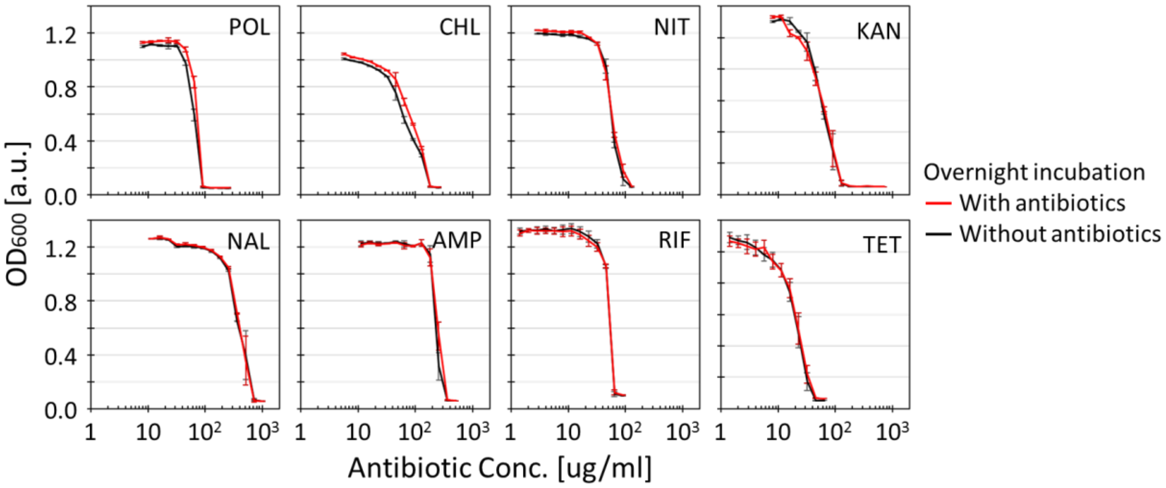


**Supplementary Fig. 10 Dose-response curves of resistant bacteria after overnight incubation with and without antibiotics.** Strains evolved under single antibiotic condition (Fig. 2) were grown for 22 hours in duplicates with and without antibiotics. For the latter case, the wild-type MIC value was used for incubation. Subsequently, they were cultured for 18 hours in increasing antibiotic concentrations. Mean values of final OD reads were used to plot the dose-response curves. Red and black curves represent the cultures with and without antibiotics, respectively. Average values of two replicates were shown in the plot with error bars (s.e.m.).

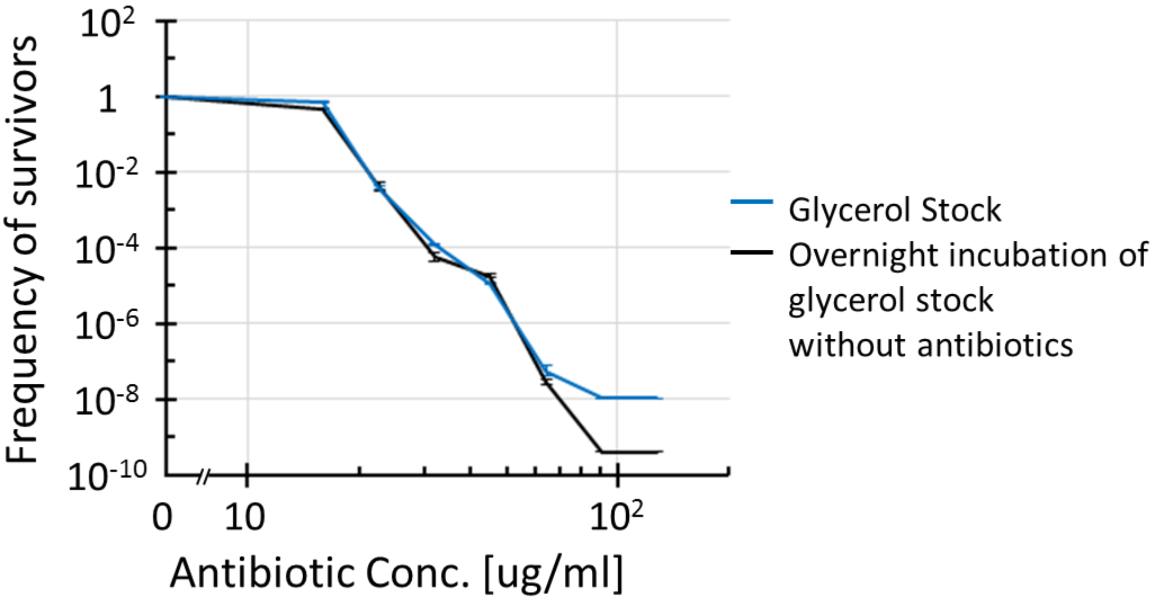


**Supplementary Fig. 11 Fraction of survivors.** The final day sample of POL and CHL cycling (Fig 4a, 24th day) which acquired only POL resistance was . The frequency of survivors was measured as colony forming unit at seven different antibiotic concentrations of POL, using a glycerol stock and overnight culture of the stock without antibiotics (shown in blue and black, respectively). No significant difference in the frequency of survivors down to $10^{-8}$ cells between the stock and the overnight culture were observed. We were not able to measure the frequency lower than $10^{-8}$ cells for the glycerol stock because the cell concentration was lower than the saturated overnight culture. Average values of two replicates were shown in the plot with error bars (s.e.m.).

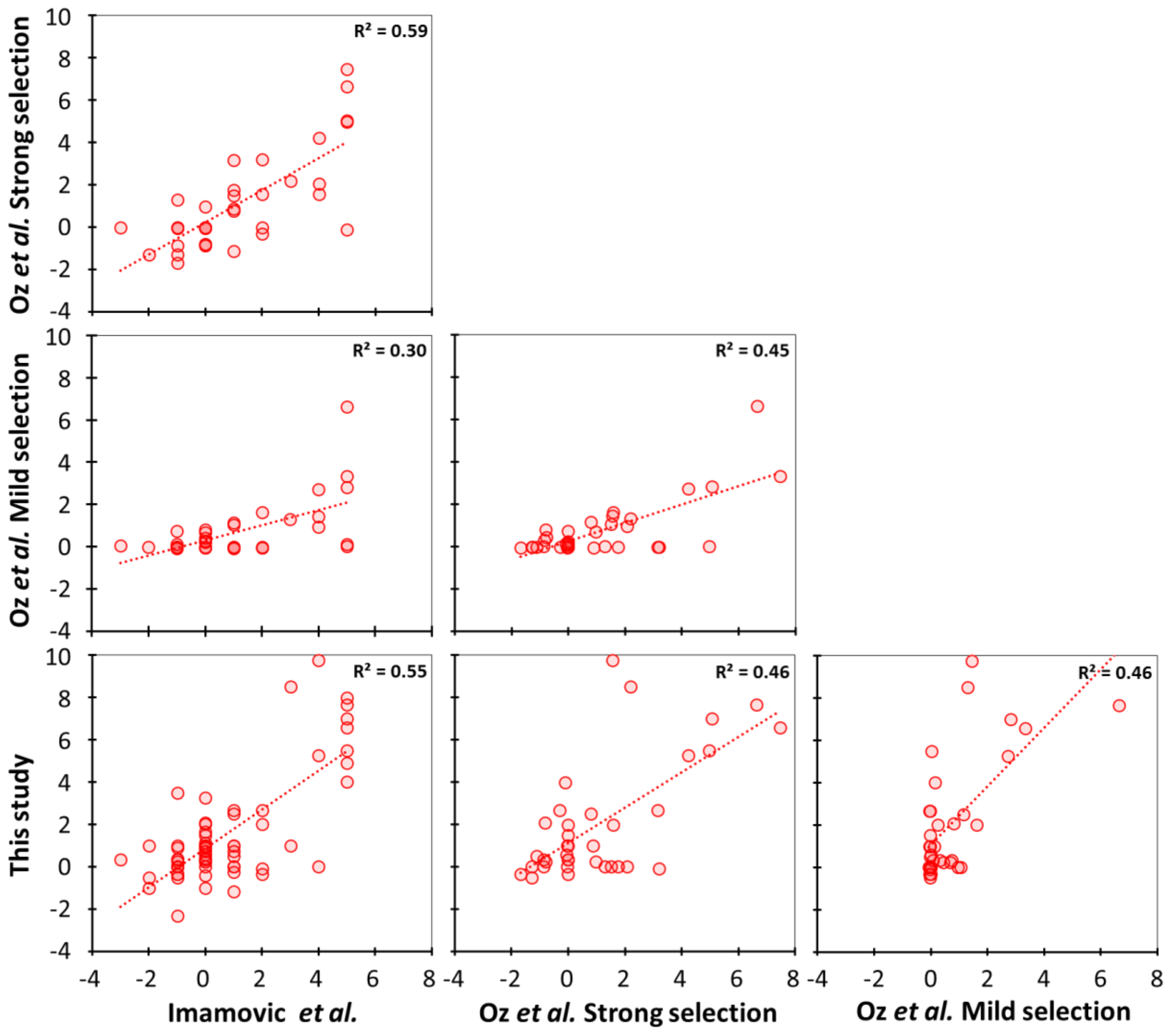


**Supplementary Fig. 12 Comparison of the $log_2$-transformed relative MIC to the parent strain.** The cross resistance/collateral sensitivity profiles between previous literature and our results were compared. Four datasets were used here, Imamovic *et al.*[1], two from Oz *et al.*[2] (strong selection and mild selection), and ours. Our dataset displayed an agreement with other datasets.

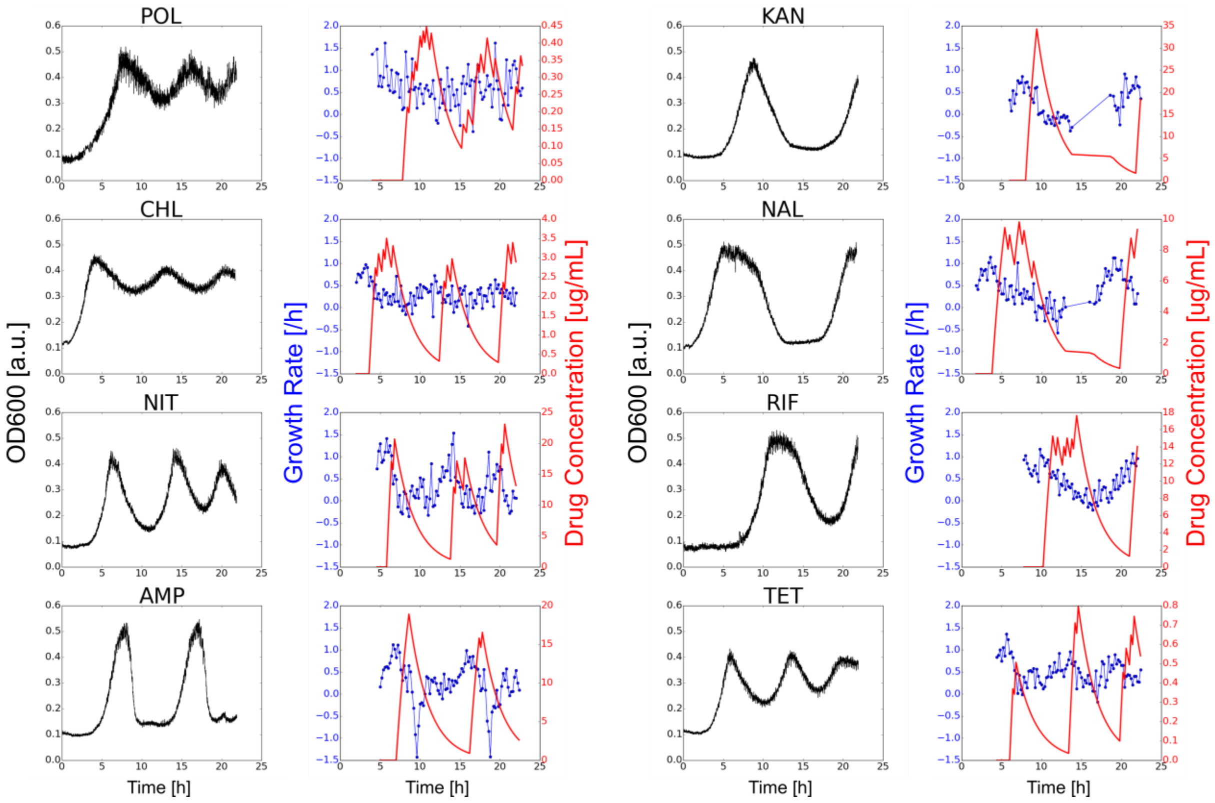


**Supplementary Fig. 13 Growth dynamics in morbidostat.** Examples of growth curves, growth rates, and drug concentration for each antibiotic are shown in black, blue and red, respectively. Antibiotics were added to the bacterial culture when OD exceeds 0.4.

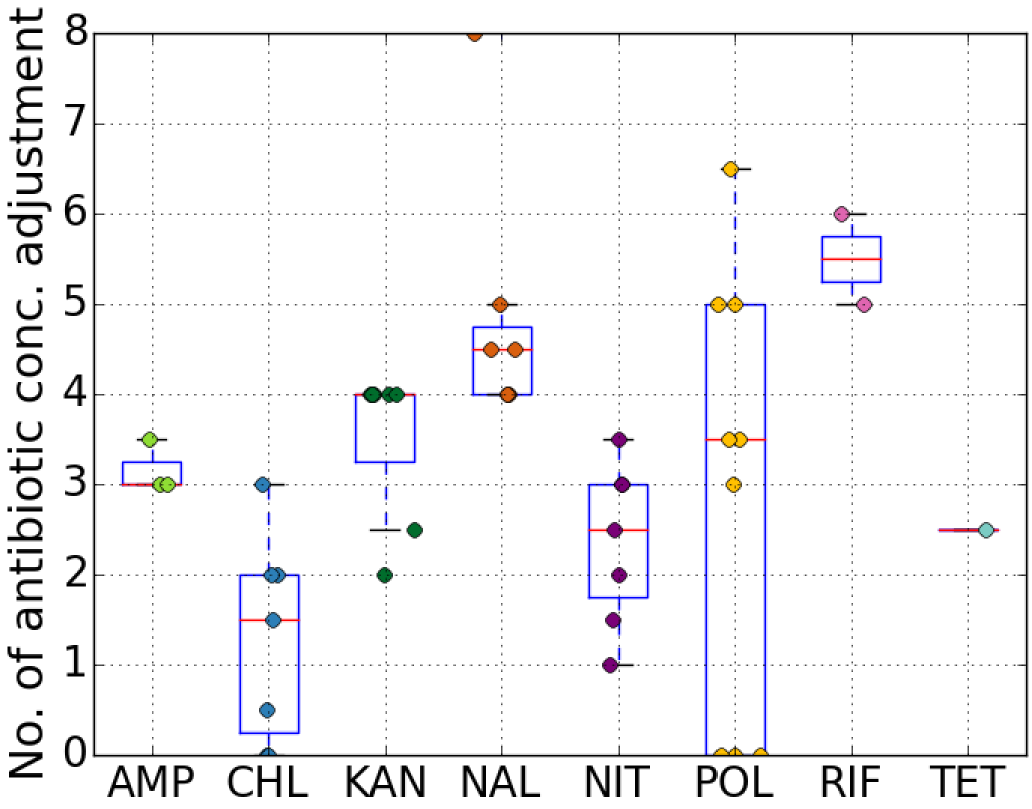


**Supplementary Fig. 14 The number of antibiotic concetration adjustments.** During the evolution experiments, the concentration of antibiotics in the drug medium was adjusted to maintain constant growth inhibition. Horizontal red bars indicate median and coloured dots are individual data used for the box plots.

## Supplementary Methods

### Theoretical model for evolution under single antibiotic condition

First, we start with an assumption that the development of bacterial resistance to antibiotic *A*, termed as $R_A$, is constantly promoted in morbidostat when antibiotic *A* is used for drug medium. This is because the system automatically adjusts the drug concentration to maintain a constant selection pressure. However, the resistance level eventually saturates due to fitness costs[3], as we observed in the morbidostat experiments (see main text). To model this, we first consider evolution under single antibiotic stress using a positive autoregulation-type equation:

$$\frac{dR_A}{dt} = \alpha_A I_A \left( \frac{R_A^n}{\theta_A^n + R_A^n} - k_A R_A \right)$$

where $\alpha_A$ is a coefficient for the rate of adaptation to antibiotic *A*, which determines how fast the antibiotic resistance increases. $I_A$ is a binary coefficient indicating the absence or presence of antibiotic *A*. When antibiotic is absent (i.e. $I_A = 0$), the resisntance does not develop. The term between the parentheses is a Hill equation-based positive autoregulation with a Hill coefficient $n$ and a delay coefficient $\theta_A$, which sets a silent period until the bacteria starts to develop the resistance for the first time. The term $k_A$ sets the maximum level of antibiotic resistance here.

By varying $\alpha_A$ and $\theta_A$, the above equation qualitatively reproduce the different patterns of bacterial evolution under single antibiotic condition in morbidostat as shown below. To determine the parameters, we first calculated fitting parameters for logistic function, $k$ (the steepness of the curve, equivalent to $\alpha_A$ here), $m$ (midpoint), and $L$ (the maximum value of the curve) for the experimental evolutionary trajectories under single antibiotic stress (Fig. 2). Based on these parameters, the duration of the silent phase $\theta$ was calculated as the time point when the unnormalised drug resistance level exceeds 0.1 for the first time (see Methods for calculation). We then used threshold values of $k = 2.5$, and $\theta = 2.5$ to classify the evolutionary patterns into three types: (1) $k > 2.5$, and $\theta > 2.5$. Larger $\theta$ delays the development of resistance and therefore gives a POL-like evolution (yellow curve in the plot below). (2) $k < 2.5$, and $\theta < 2.5$. Small $k$ and $\theta$ simulates slow and steady increase, which is similar to the resistance evolution against CHL (blue). (3) $k > 2.5$, and $\theta < 2.5$. Small $\theta$ with large $k$ gives rapid development of antibiotic resistance, similar to NAL (orange). Based

on these categorisation, we set the model parameters $\alpha_A$ and $\theta_A$ as follow (see below for determination of the system parameters):

| Name | $\theta$ | $\alpha$ | $n$ |
|---|---|---|---|
| **POL** | 0.99 | 0.4 | 2 |
| **CHL** | 0.1 | 0.1 | 2 |
| **NIT** | 0.1 | 0.1 | 2 |
| **KAN** | 0.1 | 1 | 2 |
| **NAL** | 0.1 | 1 | 2 |
| **AMP** | 0.1 | 0.2 | 2 |
| **RIF** | 0.1 | 1 | 2 |

The resistance level for each antibiotic was defined to range from 0 to 10. However, it was normalised to 0 to 1 in order to compare with experimental results, which was also normalised to 0 to 1.

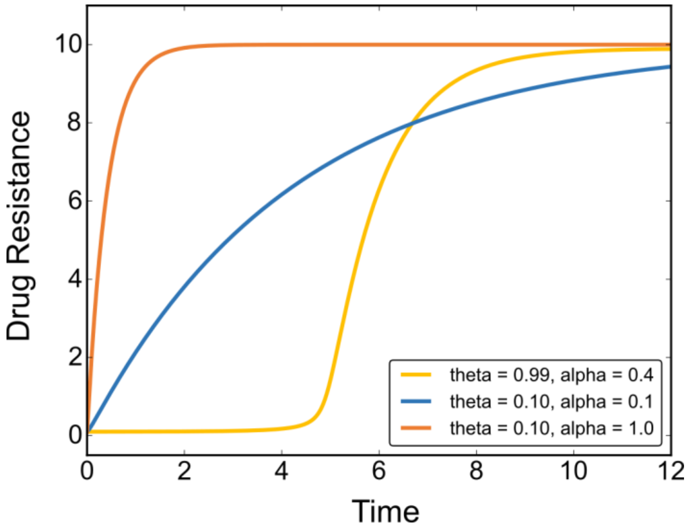


It should be noted that the above equation gives temporal evolutionary curves similar to logistic curves. Although the logistic equation $f(x) = \frac{L}{1+e^{-k(x-m)}}$ can be correlated to the above equation by $L \sim \frac{1}{k_A}$, $k \sim \alpha_A$ , and $\theta_A \sim m - \frac{\log(10L-1)}{k}$ (see Methods), we adopted this equation instead of the logistic equation because of the simplicity and symmetry with the second term as described below.

**Theoretical model for evolution using two antibiotics**

We next introduce another term for interactions with antibiotic *B*. This is introduced based on another assumption: Increased $R_A$ negatively affects resistance to another antibiotic *B*, or $R_B$, if the antibiotics are a collateral sensitive pair[1,4,5]. Otherwise it has little effect (neutral or cross resistant pair). The second term in the right hand side of the equations below is the drug interaction term, which is negative feedback loop.

$$\frac{dR_A}{dt} = \alpha_A I_A \left( \frac{R_A^n}{\theta_A^n + R_A^n} - k_A R_A \right) + \beta_{B-A} I_B \left( \frac{\theta_B^n}{\theta_B^n + R_B^n} - k_B R_A \right)$$

$$\frac{dR_B}{dt} = \alpha_B I_B \left( \frac{R_B^n}{\theta_B^n + R_B^n} - k_B R_B \right) + \beta_{A-B} I_A \left( \frac{\theta_A^n}{\theta_A^n + R_A^n} - k_A R_B \right)$$

where $\beta_{A-B}$and $\beta_{B-A}$ are the interaction strength that determines the effect of counteracting antibiotic. We empirically determined the coefficient values as $\gamma 2^{-x}$ where $\gamma$ is a scaling coefficient, x is the value from the collateral sensitivity / cross resistance profile in Fig. 2. For instance, in the case of CHL and POL cycling, $\beta_{\mathrm{POL-CHL}}$ and $\beta_{\mathrm{CHL-POL}}$ were 0.06 and 0.14, respectively, because the $MIC_{CHL}$ of the POL resistant strain and $MIC_{POL}$ of CHL resistant strain were 0.8 and -0.5 (Fig. 2). Nullclines were calculated as $dR_A/dt = 0$ and $dR_B/dt = 0$ with the condition $I_A = I_B = 1$.

**Determining system parameters**

In the current study, $\gamma = 0.1, n = 2$ and $k_A = k_B = 0.1$ were used throughout. These parameters in the model equation were determined as follows:

First, we confirmed that the evolutionary trajectories under single or multiple antibiotic conditions can be generally reproduced when the Hill coefficient was $n = 2$ or any higher integer values. However, it tended to show poor reproduciblity particularly with cycling of POL and other antibiotics when values larger than 3 were used. We speculated that this was because of the sensitivity to small changes by a larger Hill coefficient and the steep evolutionary pattern of POL resistance owing to the large $\alpha_A$. For this reason, we adopted $n = 2$ because it showed the best reproduced evolutionary patterns.

$k_A$ and $k_B$ detemine the maximum level of drug resistance a system can reach, defined as $\frac{1}{k_A}$ or $\frac{1}{k_B}$. In theory they could be any arbitrary values because the maximum resistance level is

not critical to the system's behaviour. We used $k_A = k_B = 0.1$ so that the maximum resistance level became 10 because smaller values (e.g. 1) can make the tuning of other parameters difficult. However, in principle other parameter should be tuned to reproduce similar evolutionary trajectories as the current paremter values.

To determine $\gamma$ for collateral sensitivity/cross resistance parameter $\beta$, we consider two extreme cases, $\alpha \gg \beta$ and $\alpha \ll \beta$, i.e. the cases with small and large gamma, respectively. The former case means that evolutionary trajectories under antibiotic cycling stress would be similar to those under single antibiotic stress because the first term of the equation above is a dominant factor for evolutionary patterns. In the latter case, trajectories would be sawtooth-shaped because the second term contributes to decrease in the drug resistance level during antibiotic cycling. When we look at the experimental evolutionary trajectories, however, they do not show sawtooth shapes in most of the cases. Thus we can assume $\alpha > \beta$ in the current case. Additionally, $\beta$ cannot be negative, otherwise the $\log_2$-transformed drug resistance level becomes negative. Considering $\alpha$ ranges from 0.1 to 1, we empirically determined $\gamma = 0.1$ so that $\beta$ takes values around 0.01-0.1.

**Effect of alleviated fitness cost**

As an additional adaptation process, we introduce an effect that alleviates a fitness cost incurred by a first genetic adaptation that confers bacterial resistance[6] occur. Note that this is introduced an abstract effect that cancels out the fitness cost: This could be intracellular mutations (intra-/inter-genic mutations), or population level changes (homogenous/mixed genetic population). Compensatory mutation can be an example of such effect[7]. For simplicity, we here assume drug resistant mutants with such effect have equal fitness compared to drug sensitive wild type. This effect can be incorporated in the model as negating the second term in the equations above, i.e. $\beta_{B-A} = 0$ if the bacterial population is resistant to antibiotic A with the effect. This is because the antibiotic that bacteria are resistant to has no effects whether it is present or absent. Thus, there are no selective pressure applied to lose the resistance. This reproduces evolutionary trajectories of cycling with NAL and POL, for instance (Supplementary Fig. 6b).

**Derivation of approximate analytical solutions**

Here we derive an approximate analytical solution for the equation above. We first consider the case of $dR_A/dt = 0$ with $I_A = I_B = 1$:

$$\alpha_A\left(\frac{R_A^n}{\theta_A^n + R_A^n} - k_A R_A\right) + \beta_{B-A}\left(\frac{\theta_B^n}{\theta_B^n + R_B^n} - k_B R_A\right) = 0$$

The case of $dR_B/dt = 0$ can be written similary, thus omitted here. For the antibiotics except POL, we assume $\theta_A^n \ll R_A^n, \theta_B^n \ll R_B^n$ in the current case with $n = 2$. With $k_A = k_B = k$, the equation was simplified as

$$R_A = \frac{1}{k}\left(\frac{\alpha_A}{\alpha_A + \beta_{B-A}}\right)$$

Thus, the normalised antibiotic resistance $R'_A$ can be written as

$$R'_A = \gamma R_A = k R_A = \frac{\alpha_A}{\alpha_A + \beta_{B-A}}$$

## Supplementary References


1. Imamovic, L. & Sommer, M. O. A. Use of Collateral Sensitivity Networks to Design Drug Cycling Protocols That Avoid Resistance Development. *Sci. Transl. Med.* **5,** 204ra132-204ra132 (2013).

2. Oz, T. *et al.* Strength of selection pressure is an important parameter contributing to the complexity of antibiotic resistance evolution. *Mol. Biol. Evol.* **31,** 2387–2401 (2014).

3. Lenski, R. E. Bacterial evolution and the cost of antibiotic resistance. *Int. Microbiol.* **1,** 265–70 (1998).

4. Lázár, V. *et al.* Bacterial evolution of antibiotic hypersensitivity. *Mol. Syst. Biol.* **9,** 700 (2013).

5. Suzuki, S., Horinouchi, T. & Furusawa, C. Prediction of antibiotic resistance by gene expression profiles. *Nat. Commun.* **5,** 5792 (2014).

6. Melnyk, A. H., Wong, A. & Kassen, R. The fitness costs of antibiotic resistance mutations. *Evol. Appl.* **8,** n/a-n/a (2014).

7. Hughes, D. & Andersson, D. I. Evolutionary consequences of drug resistance: shared principles across diverse targets and organisms. *Nat. Rev. Genet.* **16,** 459–471 (2015).
